# Registry Systems for COVID-19 Vaccines and Rate of Acceptability for Vaccination Before and After Availability of Vaccines in 12 Countries: A Narrative Review

**DOI:** 10.3390/idr14010016

**Published:** 2022-02-11

**Authors:** Dimitrios Papagiannis, Foteini Malli, Konstantinos I. Gourgoulianis

**Affiliations:** 1 Public Health & Vaccines Lab, Department of Nursing, School of Health Sciences, University of Thessaly, 41110 Larissa, Greece; 2 Respiratory Disorders Laboratory, Faculty of Nursing, University of Thessaly, 41110 Larissa, Greece; mallifoteini@yahoo.gr; 3 Respiratory Medicine Department, Faculty of Medicine, University of Thessaly, 41500 Larissa, Greece; kgourg@med.uth.gr

**Keywords:** vaccines, registry systems, COVID-19, acceptability

## Abstract

Registry systems play a key role in promoting vaccination campaigns in the general population. In the present narrative review, we provide data from 12 12 countries for vaccination acceptance before the availability of COVID-19 vaccines and vaccination coverage once it is available. We selected a randomized representative sample of 12 countries from WHO regions and 194 total members by the Open Epi Random Program. We observed the results with different levels of vaccine acceptability between the studies that were performed before the availability of a vaccine against COVID-19 and the vaccination coverage after the availability of the COVID-19 vaccine. All the registry systems that were developed for the recent pandemic achieved the initial functional goals. Twelve months after the vaccination campaign has begun, varying results were reported for vaccination coverage against COVID-19 vaccines with rates as high as 98% (subjects with at least one dose of vaccine) in the United Arabic Emirates, and as low as 24% in South Africa. The United Arabic Emirates stood as the leader of the world with the highest number of vaccinations 88% fully vaccinated citizens followed by Canada with 80% fully vaccinated citizens. The available data suggest that vaccine registry systems could help increase vaccination coverage and aim in the control of future outbreaks.

## 1. Introduction

Vaccination programs are complex public health interventions that offer major health benefits. Measurable of vaccination coverage is a major health indicator [1]. The recent pandemic highlighted various global problems but also stressed significant opportunities for izingreorganizing health structures and services. The experience from SARS-CoV-1 and rapid decryption in the genome of SARS-COV 2, helped in the progress of research conducted by private and public research agencies in creating a vaccine to address the pandemic [2]. Once the genetic information of SARS-CoV-2 became available, the scientists quickly selected a sequence to express the stabilized spike protein of the virus. Nine months after the start of the research, the licensing authorities approved the vaccine for general use in adults as an emergency response procedure. While mRNA technology first appeared being used for vaccine production for mass vaccination, it also gave us a significant amount of data about immunogenicity and the safety of vaccines [3,4].

Decision-makers at all levels, and especially the public health authorities, require relevant, reliable, and timely information to support the decision-making process. Information systems play a key role in providing data that guides the strategic, managerial, and operational decisions of any health initiative [5]. Moreover, immunization registries have the potential to assist health care officials in tracking and enhancing delivery for a broad range of important health services for citizens. Some Immunization registries are confidential, population-based, izedcomputerized databases that record all the immunization doses administered by volunteers and health personnel to persons residing within a specific locality [6].

Immunization registries gather immunization information with increased interest in preschool children or specific groups such as health professionals, immigrants, travelers or the elderly [7]. The development of community-based registries first began in the USA in the early 1970s. Since the early 1990s, a concerted effort has been made to develop community and state-based immunization registries [8,9].

This confidential and population-based information system contains data on vaccine doses administered. This type of system allows monitoring of vaccination coverage by service providers and includes information regarding vaccine type, dose, age, target group, and geographical area. Additionally, it yields results that facilitate individualized monitoring of immunization recipients. A fully functioning immunization registry system tracks all data of the population as they are recorded by the public health authorities such as the US Centers for Disease Control and Prevention (CDC). An immunization registry system may provide immediate access to immunization records which may serve as a safety net from losing important data in public health emergencies [6]. As of fall 2000, 24 out of 50 United states of America USA had laws or rules with three of them specifically authorizing an immunization registry. Nine additional states have laws specifically addressing the sharing of Immunization Information Systems (IIS) [8].

The European Centre for Disease Prevention and Control (ECDC) Strategy 2021–2027 defines ECDC’s goals in the upcoming years to continue ensuring that decision-makers receive the necessary advice and scientific evidence to support changes in policy and practice in the area of communicable disease prevention and control. ECDC coordinated with the European Union Member States, European Union Institutions, and several international partners, such as the World Health Organisation (WHO) Regional Office for Europe. ECDC, will continue to monitor drivers for infectious diseases, environmental conditions that contribute to the spread of disease vectors or travel pathways through which pathogens could be introduced to the EU [10]. The ECDC provides technical guidance to EU members and stakeholders for the development of an immunization information system. The functionalities of an immunization information system include personalized information on vaccination, a communication platform that allows targeted communication towards healthcare professionals and the public, decision support systems for vaccine providers (e.g., automated protocols for vaccination catch-up), and most importantly, recording of reasons for refusal of vaccination, as well as adverse events [11]. In the real world there is a significant variation in the data that every registry collects.

In Italy, the 21 regional health authorities are in charge of organizing and implementing their vaccination strategy, based on the national vaccination plan. Immunization coverage varies greatly among the regions for certain vaccines. A total of 15 regions are fully computerized, with 83% of local health units equipped with a computerized register (previously 70%). Eight of the 15 fully computerized regions use the same software, thereby, simplifying data sharing. Only four regions are able to obtain data in real-time from local health units [12].

According to the ECDC survey that was conducted in 2016, the status of implementation of vaccine registry systems in the 27 countries in Europe included three categories. The first category was Countries with IIS in place: Eight countries have a currently operating national system that meets the US-CDC definition of an IIS, namely Denmark, Finland, Iceland, Ireland, Malta, the Netherlands, Norway, and Romania. The second category was Countries with piloting IIS: Four countries, Greece, Hungary, Latvia and Slovakia are piloting a national system. Further, the last category included countries with no IIS: Six countries (Croatia, Cyprus, Czech Republic, Estonia, Luxembourg, and Slovenia) have no IIS in operation or being piloted [13].

The Australian Immunization Register (AIR) is a national registry that records all the vaccines administered to people in Australia. The 2015 AIR was Undertaken to expand the scope of Australia’s two existing immunization registers; the Australian Childhood Immunization Register and the National Human Papillomavirus (HPV) Vaccination Program Register, to improve vaccination coverage rates across the entire Australian community [14].

The IIS of Uruguay was established in 1987 to provide a computerized registry of all children born in the country and allow monitoring of their vaccination history. The system is based on the use of a single vaccination registration form. Vaccination centers, both public and private, complete one such form for each immunized child and record the doses and vaccines administered. These paper forms are submitted at the departmental level to the offices of the Honorary Commission for the Tuberculosis Campaign and Prevalent Diseases (CHLA-PE), where they enter the data into the system. Every 15 days, each region of Uruguay sends an electronic update to the national level office. They also submit paper forms or slips of children born in other departments to the respective departments where their data is entered. There is a consolidated database at the national level. Uruguay is now developing a more modern IIS that considers the information requirements of all levels [15].

In Greece, before the COVID-19 pandemic, no registry system for vaccines was available and only a piloting national system was in use. The information about the vaccination coverage for infants, adults or special groups, such as students or health care workers, came from studies and the ECDC reports [15,16,17,18,19,20].

In Israel, a child national computerized registry system was implemented in September 2008 as a pilot project in seven clinics of the Ministry of Health in each district [21].

The main targets of IIS for COVID 19 vaccines were:

1. To monitor the effectiveness of vaccines and vaccination programs, including adverse events;
2. To inform immunization policy;
3. To monitor vaccination coverage;
4. To publish the digital COVID-19 Certificate.

The aim of the present review is to provide and summarize data on COVID19 immunization registries that were developed for the recent pandemic mass immunization campaigns.

## 2. Materials and Methods

We selected a randomized representative sample of 12 countries from WHO regions and 194 total members by the Open Epi Random Program. Published articles for the present narrative review have been chosen by searching on accredited sites “PubMed, Scopus, and Google Scholar”, official government sites, and databases of organizations “such as WHO, FDA, EMA, CDC, and ECDC”. The keywords we used for each country include “registry systems for COVID-19 vaccines, acceptability of COVID-19 vaccines”. We examined also studies that recorded the acceptability of the SARS-CoV-2 vaccines before the license of the vaccines. We used mainly articles in the English language that were published in international literature or were available on governmental internet sites.

We aimed to provide data on immunization information systems that were developed for the recent pandemic mass immunization campaign against COVID-19. In addition, we examined studies that were conducted before the initiation of the vaccination campaign against COVID-19 that measured the level of the acceptability for the new vaccine in these countries.

## 3. Results

We identified 12 IIS around the world (Table 1) that address the vaccination coverage for each country against COVID-19. At the time that the manuscript was prepared the largest coverage was observed by the United Arab Emirates “UAE”, where 88% of the population is fully vaccinated followed by Canada with 76% while the lowest is recorded in South Africa with 24% of the total population being fully vaccinated.

In the present narrative review, we observed varying percentages about vaccine acceptance between studies that were performed before the availability of COVID-19 vaccines and the vaccination coverage after the availability of COVID-19 vaccines Table 2. All the registry systems that were developed for the recent pandemic achieved their initial functional goals.

For eight countries we found studies for acceptability of COVID 19 vaccine before vaccine availability. Vaccine acceptance varies according to the nationality of the study and has been reported to be 80% in the general population of Canada and 75% in Australians. For some countries like Greece the studies were conducted to special groups such as health professional. In France we found studies assessing vaccine acceptance in both, general population and health professional with a vaccine acceptance rate of 58.9% and 62% accordingly (Table 2). The vaccine registry systems were that were developed for the recent pandemic, improve the surveillance for COVID 19 vaccinations. The data are shared in real-time between the public health authorities and health system partners, and additionally analyzed by the national administration in order to respond more efficiently to the current crisis. The main functionalities of the 12 registry systems were to record citizens according to their individual insurance security number, and their vaccination status against COVID 19. The registry systems provide automated vaccination protocols and include reminders for vaccinations through telephone messages or emails. Daily or weekly data on vaccinations by sex, age group, and region are made public by ministries of health.

## 4. Discussion

The Global Vaccine Action Plan (GVAP), which was endorsed by the World Health Assembly in May 2012, reiterated several goals and principles of the Global Immunization Vision and Strategy. Additionally, it also included some shifts in the emphasis, for the development of delivery technologies and for the social science research to enhance access to computerized monitoring of the vaccination coverage and delivery of vaccines [34]. According to the CDC, one of the ways to build trust towards COVID-19 vaccines is to communicate transparently about the process of authorizing, approving, making recommendations, monitoring the safety, distributing, administration of COVID-19 vaccines, and the data handling [35]. The provider should post regular updates on benefits, safety, side effects and effectiveness; communicate the unknown. It is necessary to proactively address and mitigate the spread and harm of misinformation via social media platforms, partners, and trusted messengers.

Vaccine hesitancy is considered a top public health threat globally [36]. COVID-19 vaccine hesitancy will pose substantial risks for both the people who delay or refuse to be vaccinated and the community. The most frequently mentioned reasons for not taking the COVID-19 vaccine are the lack of confidence in the safety and efficacy of the vaccines, complacency regarding the individual risk of getting infected with COVID-19, and lack of time to get a vaccine [37,38]. All these aspects can be easily addressed by the data provided by IIS to both citizens and scientists. Among other factors, a lack of trust in political and scientific institutions has been shown to further lower vaccine acceptance [39,40] or other studies which have found that younger age is associated with being unsure or unwilling to be vaccinated against COVID-19 [41,42,43]. Study from Ireland and UK demonstrated that COVID-19 vaccine-hesitant people were distinguished from vaccine-accepting people by being more distrustful of expert’s scientists and health care professionals, and more likely to hold conspiracy theories [44].

In the present narrative review, we provide data about the vaccines registry systems that were developed during the COVID-19 period in 12 countries around the world and vaccine acceptance rates before the global vaccination campaign started and the vaccination coverage for COVID-19 after the availability of vaccines.

Health authorities of Greece approved the first SARS-CoV-2 vaccine on December 2020, and vaccines were administered first to health care workers on 27 December 2020. A survey conducted in February 2020 before the pandemic in Greece shows that 43% of Greek Health care workers were reluctant to be vaccinated against COVID-19 [45]. After the two waves of the pandemic have increased cases and deaths of COVID-19, another study from the same region showed acceptance of 78% for COVID-19 vaccination from health professionals [46]. In countries with multiple surveys, over time the following changes in COVID-19 vaccine acceptance rates were observed. In France, the vaccine acceptance rate increased from 62.0% to 77.1% in March/April and was 58.9% in June. In Italy, the vaccine acceptance rate was 77.3% in April, 70.8% in June and it reached 53.7% in September. The vaccine acceptance rate in the US was 56.9% in April, and ranged from 67.0% to 75.0% in May, and reached 75.4% in June [47].

Greece used a vaccination registry system for the first time in its history as a tool for public health policies. The main goals of the Registry System are to monitor the situation, evaluate all the available information, provide health professionals with recommendations and give an overview of its COVID-19 research, to vaccinate the entire population over 12 years of age, to monitor the effectiveness of vaccines and vaccination programs, including adverse events, to monitor vaccination coverage, to inform immunization policy and research, and EU digital COVID-19 Certificate [30]. About 1 year after the vaccination campaign began, (68%) of the total population had received at least one dose and 64% have been fully vaccinated in Greece [48].

In the recent pandemic of COVID-19, the global scientific community could observe in real-time the total progress of vaccination campaigns in each country separately. Organizations such as Oxford university with the information available on the immunizations platform “Our World in Data” and ECDC use the most recent official numbers from governments and health ministries worldwide. The population estimates, which were used to calculate per-capita metrics are all based on the latest revision of the United Nations World Population Prospects. A full list of the countries with specific sources is available to all the researchers [48,49].

Israel’s vaccination campaign began on December 2020, approximately 1 week after the FDA granted emergency approval for the vaccine (BNT162b2). On 3 December 2021, almost 12 months after the vaccination campaign began, 62% of the total population had been fully vaccinated. A previous study reported data in the age group over 90, 94.6% of the population were vaccinated, in the age group ranging from 80 to 89 years, 93% were vaccinated, in subjects 70–79 years, 95.4% were vaccinated, in the age group 60–69, years the vaccination was completed for 86.6%, and in the population aged 50–59 years, 84.6% were vaccinated. When compared, the number of vaccines administered in Israel is among the highest in the world [50]. The Ministry of Health of Israel with the COVID-19 Vaccination Information system [24] achieved the major targets of this program, which are to monitor the effectiveness of vaccines and vaccination programs (including the adverse events) to monitor vaccination coverage and to inform immunization policy and research.

A previous study that was conducted in Australia, documented the changes in vaccination rates among children following the implementation of the Australian Childhood Immunization Register (ACIR) in 1996. Following the linkage of ACIR participation with parental and provider financial incentives in 1998 and inclusion of immunizations administered overseas in 2001, reported rates for full immunization among children aged 24 months in Australia increased from 64% in 1997 to 92.7% in 2007. This study documented the association of improvements in vaccination coverage with the implementation and enhancement of the vaccines registry system of Australia [51]. A study that was conducted in Australia in June 2020, recorded an acceptance rate of 75.8% for the upcoming COVID-19 vaccine [52], which seems similar to the acceptance rate as it has been reported in recent studies. Ten months after the vaccination campaign began, 78% of the population had received at least one dose and 73% had been fully vaccinated in Australia [48].

Health authorities of Canada approved the first vaccine in December 2020, and vaccinations were administered to health care workers that same month. After a relatively slow start, Canada has achieved higher rates of both first and second vaccine doses. For instance, by December 3, 80% of the total Canadian population had received at least one dose and 76% were fully vaccinated [48]. Canada’s monitoring system for the COVID-19 vaccinations provide data about vaccination coverage and the success rate of vaccination campaign. A study was conducted by Taylor et al. before the vaccine against COVID 19 was available for use, when Canada recorded a 20% rate of vaccine refusal in Canadian residents. The respondents said that they would not get vaccinated against SARS-CoV2 although a vaccine was available. Non-adherence rates of this magnitude would make it difficult or impossible to achieve herd immunity [53]. Vaccine rejection is strongly correlated with mistrust of vaccine benefits and also correlated with worries about unforeseen future effects, concerns about commercial profiteering from pharmaceutical companies, and preferences for natural immunity [42]. In the question about the motivations to getting the vaccine the participants were most likely to report that evidence for strictly testing and safety of the vaccine were the priority [53].

The USA was the first country to authorize the COVID-19 vaccine. The vaccine has been available in the USA under Emergency Use Authorisation (EUA) since December 2020 [54]. Since COVID-19 vaccine distribution began in the United States on 14 December, many doses have been administered, thereby, fully vaccinating over 195 million people or 59% of the total U.S. population [48]. An online survey that was conducted by Reiter et al. in adults in the USA in May 2020 reported that 69% of participants were willing to get a COVID-19 vaccine. If the recommendation for vaccination were coming from health care providers participants of the study were more likely to be willing to get the vaccine [55]. A cross-sectional study conducted in the United States by Mennito et al. evaluated the association between the use of an Information Immunization System and the likelihood of children being up-to-date with their vaccines. The study demonstrated that practice policies between 2004 and 2006 that used an IIS did not have significantly higher coverage levels than those practices not using an IIS [56]. It is important, that health authorities accurately and repeatedly inform the public about the evidence on the safety of vaccines, and address some of their salient concerns. The IIS of USA has the page for monitoring the safety of licensed and authorized vaccines for COVID 19 and conducts high-quality vaccine safety research. The vaccine safety articles and studies listed on this page include a full citation, a short summary, and a link to the free PMC article, when available [57]. The transparency of data about on safety and effectiveness of COVID-19 vaccines could be reduce the majority of concerns of the people who refuse to get the vaccine for these reasons. The aforementioned data information was freely available to the web site of CDC.

A study among General Practitioners from France showed markedly different perceptions of vaccination controversies [58]. In France from 4 January, healthcare workers aged over 50 years received the BNT162b2 vaccine. Before February, the first vaccination centers started vaccinating people aged 75 years, followed by those aged 65 years. Initially, this stage of the vaccination roll-out was planned for the end of January and this led to criticism that France is vaccinating too slowly. Before the initiation of the vaccination campaign in France there was an acceptance rate for the COVID-19 vaccine of 58.9% [59]. Twelve months after the vaccination campaign began, 77% of the population have been vaccinated with the first dose, and 70% have been fully vaccinated [48].

A systematic review by showed that the Middle East was among the regions with the lowest COVID-19 vaccine acceptance rates globally. The acceptance rate was the lowest in Kuwait (23.6%), followed by Jordan (28.4%), Saudi Arabia (64.7%), and Turkey (66.0%) [47]. The UAE (at the time the present manuscript was prepared) was the world’s most vaccinated nation with 88% fully vaccinated citizens, while 98% of the total population had received at least one dose [48]. The UAE has a comprehensive, government-funded health service as well as private health facilities that deliver a high standard of care to the population. The UAE became the first country in the world to approve China’s Sinopharm vaccine for widespread use in early December 2020. The government has also approved the BNT162b2, ChAdOx1-s and Sputnik V vaccines all of which are dispensed free to residents.

Uruguay, started its inoculation campaign in March 2021 with a primary focus on teachers, soldiers, police and firefighters. At this time according to the “Our World in Data” Uruguay recorded high level of vaccination coverage against the COVID-19 vaccine with 76% percentage of the population fully vaccinated [48]. Studies from South America showed an acceptance rate for Ecuador of 71.9% and Mexico of 76.3% before the Global vaccination campaign started [60]. The main targets for the Informatics system of Uruguay were to vaccinate the entire population over 12 years of age, to monitor the effectiveness of vaccines and vaccination programs, including adverse events, to monitor vaccination coverage, to inform immunization policy and research, and provide digital COVID-19 Certificate.

In Italy the first doses of the vaccine were administered on 31 December 2020. Priority was given to healthcare professionals, elderly residents, and personnel in care facilities. The acceptance rate for COVID-19 vaccine was estimated at 70.8% [47,59]. On 3 December 2021, 78% of people have been vaccinated with the first dose, and 73% have been fully vaccinated [48]. The main targets for the Informatics were to vaccinate the entire population over 12 years of age, to monitor the effectiveness of vaccines and vaccination programs, including adverse events, to monitor vaccination coverage, to inform immunization policy and research, and EU digital COVID-19 Certificate [23].

The German government was criticized for the sluggish start to its vaccination campaign but it has recovered quickly. Ten months after the vaccination campaign began, 71% of people have been vaccinated with the first dose, and 68%. have been fully vaccinated in Germany [48]. The acceptability of COVID 19 vaccines in the general population is over 68% [47]. The Robert Koch Institute COVID-19 in Germany developed an effective monitoring system for COVID-19 vaccination campaign with the main goals as to monitor the situation, evaluate all available information, estimate the risk for the population in Germany, provide health professionals with recommendations and give an overview of its COVID-19 research, with a weekly situation report, including epidemiological data, risk assessment, numbers of tests for SARS-CoV-2, vaccination monitoring, intensive care capacities and more. Additionally, it provided a dashboard with notified case numbers by state (Bundesland) and district (Landkreis, in German) and the last EU digital COVID-19 Certificate [31].

The confirmation of first case in Japan were done on 16 January 2020, in area of central coast of Japan’s island, Honshu. The first infection was detected in Japan on time by the implementation active surveillance. In the first pandemic wave there were no strictly quarantine measures [60]. Acceptance rate was estimated at 65.7% of the elderly residents of rural areas; those with underlying conditions and males (vs. females) demonstrating higher acceptability towards vaccination [61]. Others have observed that 62.1% of the population would likely vaccinate against COVID-19 [62]. Vaccine rollout had been slow in Japan compared with other countries. Japan provided vaccines to (first to healthcare workers, followed by elderly) and the campaign started on 17 February 2021 [63]. Japan developed a monitoring system for COVID-19 vaccinations by the Ministry of Health. The main targets of the information system are to monitor the effectiveness of vaccines and vaccination programs, including adverse events, to monitor vaccination coverage and to inform immunization policy [26]. Japan became the second country with the highest percentage of vaccination coverage against COVID-19. On 3 December 2021, 79% of people had been vaccinated with one dose, and 77% had been fully vaccinated [48].

On 3 December 2021, the country with the lowest vaccination coverage among the 12 countries examined was South Africa with only 24% of the total population fully vaccinated. Vaccine hesitancy is, however, neither new nor unique to COVID-19 vaccines, the recent global outbreaks of vaccine-preventable diseases, such as the various measles outbreaks in the United States of America (USA) and Europe, have been largely attributed to vaccine hesitancy [64,65,66,67]. Vaccine hesitancy in a South Africa was challenge faced by the personnel in vaccination programs in a study conducted in 2009 [68]. The inherently societal nature towards COVID-19 and hesitancy to vaccinate, potentially influenced by age, race, education, politics, geographical location, and employment [69]. South Africa began its national COVID-19 vaccination program in February 2021. The vaccination campaign targeted healthcare workers, prioritizing essential workers, people older than 60 years, adults with co-morbidities, and people in congregate settings, [69,70]. The initial goal was to vaccinate a minimum of 67% of the 60 million population to achieve herd immunity. Eight months after the vaccination campaign began, 29% of people have been vaccinated with at least one dose, and 24% have been fully vaccinated [48]. The monitoring system was developed by the Ministry of Health. The main targets are to identify eligible vaccination beneficiaries to plan the supply of vaccines to allocate beneficiaries to their nearest available service point and to provide information about the vaccination program about the vaccination program, including (eligibility, and follow-up) [35].

In many places, the pandemic continues unabated. Some countries are currently suffering their highest rates of hospitalization and death. Additionally, even in areas where it has subsided, the end point continues to recede into the future. Many countries continue with low vaccination coverage. It is perfectly understandable that vaccine registry systems help the governments to record vaccination rates. This could be useful to international organizations such as the WHO for applying pressure to deliver more vaccine doses to countries with low vaccination coverage. Many countries have been inserting the third dose for COVID-19 while others have not completed the initial goals of vaccination [57]. Even more countries are at risk of missing the WHO targets of vaccinating 40% of the population of every country by the year 2021, and 70% by the middle of 2022 year [71]. It is very clear that the health crisis by SARS-COV 2 is a global epidemic and not endemic. Safe and effective of COVID-19 vaccines were developed in record time. However, the virus is moving faster than the global distribution of vaccines.

## 5. Conclusions

In the largest global mass immunization program against COVID-19, it seems to be that the computerized monitoring systems help to record the vaccination coverage globally. Countries that used computerized database vaccines registry systems recorded in real-time the vaccination coverage against COVID-19. In the present narrative review, we observed differences concerning vaccine acceptability and coverage between studies, especially when performed before and after the availability of COVID-19 vaccines. All the registry systems that were developed for the recent pandemic achieved the initial functional goals. It is time that the link between the vaccination coverage data, the data for safety and effectiveness of vaccines, and the complete data that vaccines registry systems could offer, be managed by the internationally recognized health authorities such as the WHO, CDC, ECDC, and cooperate with national health authorities. In that way, they could help increase vaccination coverage and gain control of the outbreaks. The global crisis caused by the current pandemic may be a crucial opportunity to create registry systems for vaccination coverage for vaccine-preventable diseases, the legislation on the protection of citizens’ data.

## Figures and Tables

**Table 1:** Monitoring information systems for COVID 19 vaccinations.

Country	Monitoring System	Targets of Information Immunization System:	Ref
Australia	Australian Immunization Register (AIR	-To monitor the effectiveness of vaccines and vaccination programs, including adverse events; -To identify any parts of Australia at risk during disease outbreaks; -To inform immunization policy and research; -As proof of vaccination for entry to child care and school, and for employment purposes; -To monitor vaccination coverage across Australia.	[22]
Italy	Ministero della Salute. Novel coronavirus.	-To monitor the effectiveness of vaccines and vaccination programs, including adverse events; -To monitor vaccination coverage; -To inform immunization policy and research; -EU digital COVID-19 Certificate.	[23]
Israel	Ministry of Health Coronavirus. COVID-19 Vaccination Information	-To monitor the effectiveness of vaccines and vaccination programs, including adverse events; -To monitor vaccination coverage; -To inform immunization policy and research.	[24]
Canada	Government of Canada Vaccines for COVID-19: How to get vaccinated.	-To monitor the effectiveness of vaccines and vaccination programs, including adverse events; -To monitor vaccination coverage; -To inform immunization policy and research.	[25]
Japan	Ministry of Health, Labour and Welfare. Novel Coronavirus (COVID-19). COVID-19 Vaccine Navi.	-To monitor the effectiveness of vaccines and vaccination programs, including adverse events; -To monitor vaccination coverage; -To inform immunization policy and research.	[26]
Uruguay	Ministerio de Salud Pública. Agendate y accedé a toda la información sobre las vacuna. Plan de Vacunación COVID-19.	-To monitor the effectiveness of vaccines and vaccination programs, including adverse events; -To monitor vaccination coverage; -To inform immunization policy and research; -Digital COVID-19 Certificate.	[27]
UAE	UAE CORONAVIRUS (COVID-19) UPDATES National Emergency Crisis and Disaster Management Authority.	-The goal is to vaccinate the entire population; -To monitor the effectiveness of vaccines and vaccination programs, including adverse events; -To monitor vaccination coverage; -To inform immunization policy and research.	[28]
USA	CDC Vaccines for COVID-19	-Inventory Reporting Required for all providers; -COVID-19 vaccination providers will report on-hand COVID-19 vaccine inventory each day; -Increase access to COVID-19 vaccines; -Optional for providers; -CDC will be directing the public to use Vaccine Finder to find locations offering COVID-19 vaccine; -To monitor the effectiveness of vaccines and vaccination programs, including adverse events.	[29]
Greece	National Vaccination Campaign. COVID-19 Vaccination.	-To monitor the effectiveness of vaccines and vaccination programs, including adverse events; -To monitor vaccination coverage; -To inform immunization policy and research; -EU digital COVID-19 Certificate.	[30]
Germany	Robert Koch Institute COVID-19 in Germany	-Monitoring the situation, evaluating all available information, estimating the risk for the population in Germany, providing health professionals with recommendations and gives an overview of its own COVID-19 research; -Weekly situation reports, incl. epidemiological situation, risk assessment, test numbers, vaccination monitoring, intensive care capacities and more; -Dashboard with notified case numbers by state (Bundesland) and district (Landkreis, in German); -EU digital COVID-19 Certificate	[31]
France	Republique Francaise L’information fiable, utile pour votre santé.	-To monitor the effectiveness of vaccines and vaccination programs, including adverse events; -To monitor vaccination coverage; -To inform immunization policy and research; -EU digital COVID-19 Certificate.	[32]
South Africa	Republic of South Africa, Department of Health. Electronic Vaccination Data System (EVDS) Self Registration Portal.	-Identify eligible vaccination beneficiaries; -Plan supply of vaccines and ancillary items; -Allocate beneficiaries to their nearest available service point; -Communicate with enrolled individuals about the vaccination program, including but not limited to: -Eligibility; -Where they will be vaccinated and follow-up vaccination appointments.	[33]

**Table 2:** Acceptability of COVID-19 vaccine by country before vaccine availability and Vaccination coverage on 3 December 2021.

Country	Acceptance before Vaccine Availability	Study Population	Vaccination Coverage after Vaccine Availability in General Population
Australia	75.8%	General population	73%
Canada	80%	General population	76%
USA	69%	General population	59%
Italy	70.8%	General population	73%
Germany	70%, 68.4%	General population	68%
Japan	65.7%, 62.1%	General population	77%
Greece	43%, 78%	Health Professionals	64%
France	58.9%, 62.0%	General population and Health Professionals	70%

## Data Availability

Not applicable.
